# Near-Infrared Spectroscopy to Predict the Course of Necrotizing Enterocolitis

**DOI:** 10.1371/journal.pone.0154710

**Published:** 2016-05-16

**Authors:** Trijntje E. Schat, Maarten Schurink, Michelle E. van der Laan, Jan B. F. Hulscher, Christian V. Hulzebos, Arend F. Bos, Elisabeth M. W. Kooi

**Affiliations:** 1 University of Groningen, University Medical Center Groningen, Beatrix Children’s Hospital, Division of Neonatology, Groningen, the Netherlands; 2 University of Groningen, University Medical Center Groningen, Department of Surgery, Division of Pediatric Surgery, Groningen, the Netherlands; University of Chicago, UNITED STATES

## Abstract

**Objectives:**

To investigate whether cerebral, liver, and infraumbilical regional tissue oxygen saturation (rSO_2_) and fractional tissue oxygen extraction (FTOE) could be used to diagnose necrotizing enterocolitis (NEC) and complicated NEC (Bell’s stage 3B or death) during its early stages.

**Methods:**

A prospective observational cohort study of preterm infants with suspected or diagnosed NEC. We compared the mean eight-hour cerebral, liver, and infraumbilical rSO_2_ and FTOE values of infants with no NEC and definite NEC and of infants with uncomplicated and complicated NEC in the first forty-eight hours after onset of symptoms, suspicious for NEC. Furthermore, we determined cut-off values by generating receiver operating characteristics curves in case of significant differences in the first eight-hour mean values of rSO_2_ between infants with no NEC and definite NEC and between infants with uncomplicated and complicated NEC.

**Results:**

We included 33 patients: 13 no NEC, 10 with uncomplicated NEC, and 10 with complicated NEC. We found no significant differences in the first twenty-four hours after onset of symptoms in rSO_2_ and FTOE between infants with no NEC and definite NEC. In preterm infants with complicated NEC, we observed significantly lower cerebral, liver, and infraumbilical rSO_2_ and higher FTOE within twenty-four hours after onset of symptoms compared with infants with uncomplicated NEC. A continuous cerebral rSO_2_ ≤ 71% and liver rSO_2_ ≤ 59% in the first eight hours after onset of symptoms predicted the onset of complicated NEC with a sensitivity of 1.0 and specificity of 0.8, and a sensitivity of 1.0 and specificity of 1.0, respectively.

**Conclusions:**

By measuring the cerebral and splanchnic oxygenation it is possible to differentiate complicated NEC from uncomplicated NEC. In our sample, NIRS monitoring did not proof useful for distinguishing between definite NEC and no NEC in preterm infants with clinical signs suspicious of NEC.

## Introduction

Necrotizing enterocolitis (NEC) is the most devastating gastrointestinal disease in the neonatal intensive care unit. It is associated with detrimental short-term and long-term outcomes, including high mortality rates and impaired neurodevelopmental outcome [1, 2]. Currently, we lack the tools and tests to reliably diagnose NEC in its early stage and to predict its progression to becoming a complicated disease (i.e. perforated bowel corresponding to Bell’s stage 3B, or death).

Near-infrared spectroscopy (NIRS) might be a useful bedside tool to diagnose the earliest stages of NEC. In previous studies it was found that NIRS measurements differed between preterm infants with and without bowel ischemia [3–5]. NIRS measures regional tissue oxygen saturation (rSO_2_) non-invasively. Using simultaneous measurements of arterial oxygen saturation (SpO_2_), fractional tissue oxygen extraction (FTOE) can be calculated [6]. It reflects the balance between oxygen supply and consumption in tissue and can, therefore, be used as an indicator of inadequate tissue perfusion and oxygenation [6]. Since bowel ischemia seems to be strongly associated with the development of NEC, complicated NEC in particular [7], measuring splanchnic tissue oxygen saturation and extraction might help the clinician to diagnose NEC from its earliest stage onward.

Our first aim was to investigate, at the onset of the disease, the diagnostic value of splanchnic NIRS monitoring to discriminate between definite NEC (Bell’s stages 2 & 3) and no NEC (Bell’s stage 1 at most). Our second aim was to determine whether splanchnic NIRS monitoring could be used to discriminate between infants with definite NEC that would develop without complications (uncomplicated NEC) and infants with NEC that would develop with complications (complicated NEC). The latter was defined as the infant developing a bowel perforation requiring surgery (Bell’s stage 3B), or death. We hypothesized that as a result of hypoxic and/or necrotic intestinal tissue splanchnic rSO_2_ values would be lower and splanchnic FTOE values would be higher in preterm infants who developed (complicated) NEC.

## Methods

### Patient population

Between October 2010 and October 2012 we conducted a prospective observational cohort study in the neonatal intensive care unit of University Medical Center Groningen, a tertiary referral center. The study was registered in the Dutch Trial Registry under number NTR3239. We included preterm infants without abdominal wall defects who were suspected of having NEC or who had already been diagnosed with NEC. Abdominal radiographs were made as soon as possible after suspicion of NEC; the diagnosis was confirmed if pneumatosis intestinalis was present. The modified Bell’s staging criteria were used for diagnosis [8]. In case of definite NEC (minimal Bell’s stage 2), our protocol indicates that sequential abdominal radiographs be taken every eight to twelve hours until it is evident that radiographic signs of NEC have resolved and clinical signs have stabilized.

Written informed parental consent was obtained in all cases. The study was approved by the institutional ethics review board of University Medical Center Groningen.

### Near-infrared spectroscopy

We used the INVOS 5100C near-infrared spectrometer (Covidien, Mansfield, MA, USA) in combination with the neonatal SomaSensors (Covidien) to measure oxygen saturation values of cerebral tissue and in the splanchnic region. We placed the SomaSensors to the left or right frontoparietal side of the infant’s head to measure cerebral tissue oxygen saturation (r_c_SO_2_). The oxygen saturation of the splanchnic region was measured at two abdominal locations: below the right costal arch to measure liver tissue oxygen saturation (r_liv_SO_2_), and infraumbilically on the central abdominal wall to measure intestinal tissue oxygen saturation (r_int_SO_2_). The sensors were held in place by elastic bandaging or Mepitel (Mölnlycke, Sweden) and were only removed temporarily during routine nursing care, clinical assessments, and radiographic examinations. Afterwards, they were replaced onto the same location. NIRS monitoring started as soon as possible after NEC was suspected or diagnosed and was continued for forty-eight hours. Simultaneously, we measured SpO_2_ and calculated FTOE with the equation: FTOE = (SpO_2_ –rSO_2_) / SpO_2_ [6].

We previously reported on the feasibility and safety of monitoring oxygenation in both the liver and infraumbilical region and the correlation and agreement between these measurements [9]. The infants reported in that article are also part of the study group described in this manuscript. However, we did not report any findings concerning the course of rSO_2_ and FTOE values in relation to the development of definite NEC and complicated NEC.

### Clinical variables

Prospectively, we collected neonatal characteristics including gestational age, postnatal age at first NIRS measurement, birth weight, and gender. Furthermore, we documented the presence or absence of anemia (defined as a hemoglobin level < 8.0 mmol/L), thrombocytopenia (defined as a platelet count < 150 x 10^9^/L), and metabolic acidosis (defined as pH < 7.30 and HCO_3_^-^ < 22 mmol/L) within twenty-four hours before and twenty-four hours after onset of NEC symptoms. Furthermore, we registered signs of circulatory failure and patency of the ductus arteriosus (determined by echocardiography) during the first forty-eight hours after onset of NEC symptoms, and treatment for a patent ductus arteriosus before the onset of NEC symptoms. Circulatory failure was defined as hemodynamic instability and scored by the need for volume expansion or the use of inotropes or both, from one hour before onset of NEC symptoms until the first forty-eight hours after onset, or until surgery, whichever came first.

Onset of NEC symptoms was defined as the time of the first abdominal radiographic examination after clinical suspicion of NEC, including the radiographs done in the referring hospitals. After completion of the study a panel of four experts (MS, JBFH, AFB, EMWK), blinded for the NIRS measurements, classified the infants independently of one another into modified Bell’s stages using clinical and radiological parameters. For those infants who had been classified differently by the individual panel members the final Bell’s stage was determined by consensus.

To address our first aim, we classified the infants into two groups: infants with no NEC (Bell’s stage 1 at most) and infants with definite NEC (Bell’s stages 2 & 3). For our second aim, we analyzed, in infants with definite NEC, the differences between infants with uncomplicated NEC and infants with complicated NEC (Bell’s stage 3B, or death as a consequence of NEC).

### Statistical analysis

We used SPSS 22.0 software for Windows (IBM SPSS Statistics 22, IBM Corp., Armonk, New York, USA) for the statistical analyses.

R_c_SO_2_, r_liv_SO_2_, and r_int_SO_2_ values were recorded by the INVOS 5100C every six seconds. SpO_2_ was registered every five minutes. We then matched one rSO_2_ value that corresponded time wise to every SpO_2_ value, leaving one measurement per five minutes for rSO_2_ and SpO_2_. Next, we constructed six eight-hour periods starting from onset of NEC symptoms (first abdominal radiographic examination) and calculated eight-hour means of r_c_SO_2_, r_liv_SO_2_, r_int_SO_2_, cerebral FTOE (cFTOE), liver FTOE (livFTOE), and intestinal FTOE (intFTOE) values. Means of rSO_2_ and FTOE values were only used for further analyses if they were based on at least 30 minutes of available values. We also constructed eight one-hour periods, starting from onset of NEC symptoms, and calculated one-hour means of r_c_SO_2_, r_liv_SO_2_, r_int_SO_2_, cFTOE, livFTOE, and intFTOE values during these first eight hours after onset of NEC symptoms.

The Mann-Whitney test was used to compare the eight-hour and one-hour mean values of rSO_2_ and FTOE between infants with no NEC and definite NEC and between infants with uncomplicated NEC and complicated NEC. We repeated the analyses with only infants who were included within eight hours after onset of disease, and in whom NIRS measurements were available at all three locations. Next, a receiver operating characteristics (ROC) curve was constructed of the rSO_2_ values that were statistically significantly different between groups in the first eight hours after onset of NEC symptoms to assess sensitivity and specificity and to determine potential cut-off values to predict the development of definite and complicated NEC within forty-eight hours after onset of NEC symptoms.

Finally, we determined the variability of the r_c_SO_2_, r_liv_SO_2_, and r_int_SO_2_ measurements separately during the first forty-eight hours after onset of NEC symptoms, by calculating each infant’s daily intraindividual variability, defined as the daily percentage of time that one-hour mean rSO_2_ values were 15% or more below or above the infant’s daily mean [10].

To test whether there were differences between groups we used the chi-square test or Fisher exact test for categorical data and the Mann-Whitney test for continuous data. A *P* value of < .05 was considered statistically significant.

Since this study was of an exploratory nature, we refrained from performing extensive statistical analyses. Because clinical and radiographic symptoms and signs are evaluated every eight to twelve hours in our neonatal intensive care unit, we decided to calculate 8-hour mean values of NIRS measurements in order to investigate the possibility of using rSO_2_ and FTOE values in routine clinical care.

## Results

**Fig 1:**
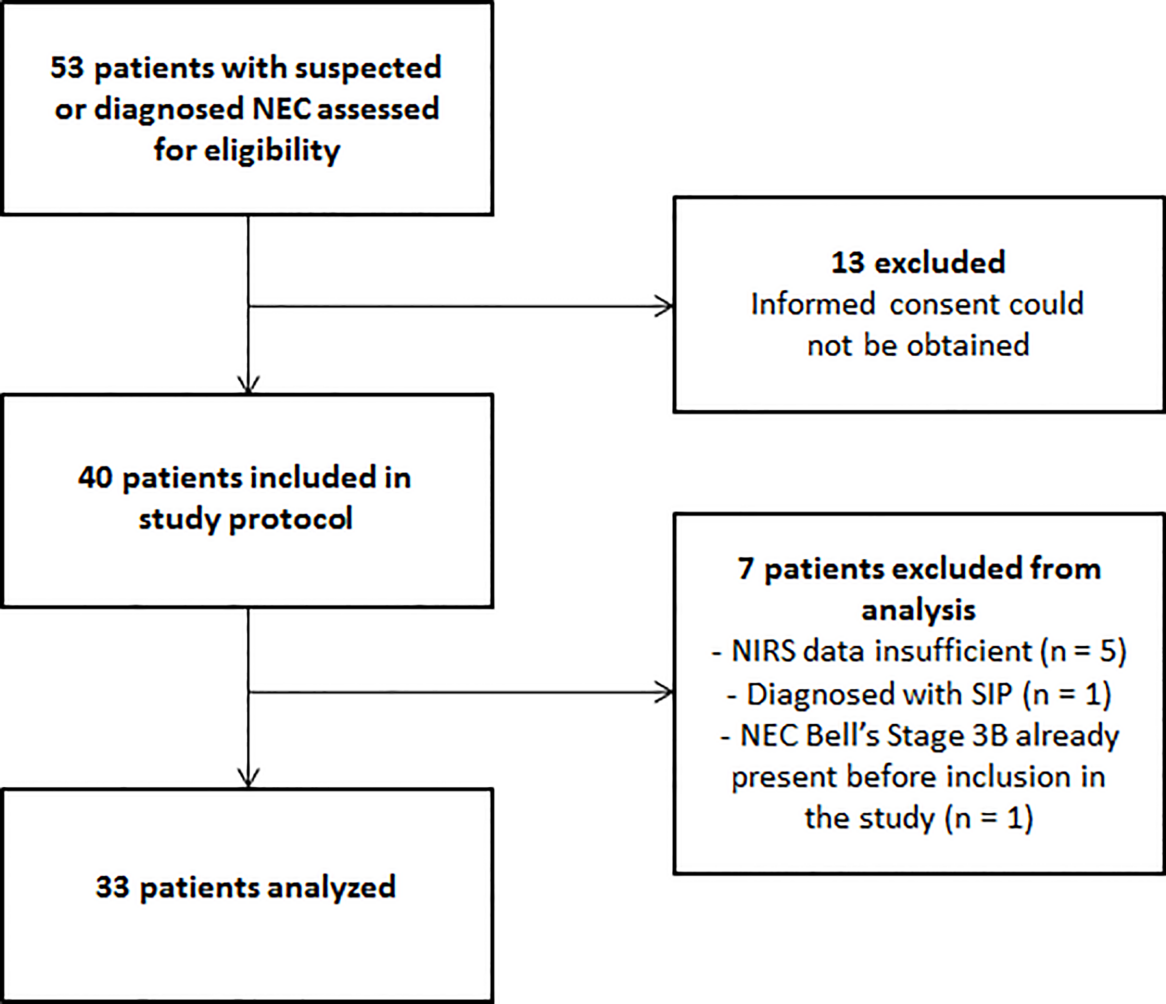
Flow diagram of the study.

A total of thirty-three infants were included for final analysis (Fig 1). Gestational age ranged from 25 to 35.9 weeks (median 28.3 weeks), birth weight ranged from 570 to 2400 grams (median 1235 grams). NIRS monitoring commenced after a median of seven hours (range 1–32) after onset of NEC symptoms. We were able to measure 24 infants at all sites including r_liv_SO_2_ and r_int_SO_2_. In 15 of these 24 infants measurements started before eight hours after onset of NEC symptoms, although in two of them measurements within the first eight hours were too short to yield reliable values. In seven infants we were unable to measure r_liv_SO_2_: in three infants due to shortage of equipment (one infant from each group), and in four infants due to simultaneous inclusion in another multisite NIRS study in which renal rather than liver oxygen saturation was measured (two infants with no NEC and two infants with complicated NEC). In two infants we were unable to measure r_int_SO_2_: in one infant because we could not place the sensor due to the presence of an umbilical venous catheter taped to the infraumbilical skin, and in the other infant due to shortage of equipment (one infant with uncomplicated NEC, and one infant with complicated NEC).

We were able to calculate eight-hour mean r_c_SO_2_ values for 156, r_liv_SO_2_ for 115, and r_int_SO_2_ for 135 time periods out of the possible 198 (6 x 33 time periods). Median (range) time of available r_c_SO_2_, r_liv_SO_2_, and r_int_SO_2_ values every eight hours was 450 (35–480), 370 (50–480), and 375 (30–480) minutes, respectively.

### NIRS measurements in infants with no NEC and definite NEC

Twenty infants developed Bell’s stages 2 or 3 and thirteen infants ultimately did not have NEC. Clinical diagnoses of the infants without NEC were sepsis (n = 3), delayed passage of meconium (n = 2), bloody stools of unknown cause (n = 2), gastroenteritis (n = 2), sigmoid volvulus (n = 1), CPAP belly (n = 1), and abdominal symptoms of unknown cause (n = 2). Table 1 contains the patient characteristics of infants with no NEC and definite NEC. Infants with definite NEC underwent surgery significantly more often and had a higher mortality rate than infants with no NEC. Additionally, we found a trend towards a higher prevalence of anemia in preterm infants with no NEC than in infants with definite NEC.

**Table 1:** Patient characteristics of infants with no NEC and definite NEC. Data are expressed as median (range) or as numbers unless specified otherwise. Abbreviations: NEC—necrotizing enterocolitis; NIRS—near-infrared spectroscopy; PDA—patent ductus arteriosus; PNA—postnatal age; RBC—red blood cell. Circulatory failure was defined as hemodynamic instability and scored by the need for volume expansion or the use of inotropes or both, from one hour before NEC onset until the first forty-eight hours after NEC onset, or until surgery, whichever came first. Statistical differences between the two groups are marked by * (< .05).

	No NEC (n = 13)	Definite NEC (n = 20)
Gestational age (weeks)	28.3 (27.0–31.7)	28.2 (25.0–35.9)
Birth weight (grams)	1190 (570–1690)	1333 (740–2400)
Male:Female	5:8	14:6
PNA at first NIRS measurement (days)	13 (4–36)	10 (3–41)
Anemia (%)	8 (62)	6 (30)
Thrombocytopenia (%)	1 (8)	5 (25)
Metabolic acidosis (%)	2 (17) (n = 12)	3 (16) (n = 19)
Mechanical ventilation (%)	3 (23)	6 (30)
Treated PDA before onset study (%)	2 (15)	5 (25)
PDA during study (%)	4 (31)	5 (25)
Hemodynamically significant	3	2
RBC transfusion (%)	4 (31)	7 (35)
Circulatory failure		
Fluid resuscitation (%)	5 (38)	12 (60)
Inotropes (%)	0 (-)	6 (30)
Surgery (%)	0 (-)	9 (45)*
Death (%)	0 (-)	7 (35)*

In Table 2 we present the courses of rSO_2_ and FTOE values. We found no significant differences between the two groups in the first twenty-four hours after onset of NEC symptoms. From twenty-four hours onwards, however, preterm infants with definite NEC had significantly higher median r_int_SO_2_ values than infants with no NEC. Furthermore, median intFTOE was significantly lower between thirty-two and forty-eight hours after onset of NEC symptoms in preterm infants with definite NEC than in infants with no NEC. These findings did not change when we analyzed only those infants who were included within eight hours after onset of NEC symptoms and in whom NIRS measurements were performed at all three locations (S1 Table).

**Table 2:** RSO_2_ and FTOE values in the first forty-eight hours after onset of NEC symptoms in preterm infants with no NEC and definite NEC. Data are expressed as median values with the number of infants studied between brackets. Statistical differences between the two groups are marked by * (< .05).

Hours	r_c_SO_2_		r_liv_SO_2_		r_int_SO_2_		cFTOE		livFTOE		intFTOE	
	no NEC	dNEC	no NEC	dNEC	no NEC	dNEC	no NEC	dNEC	no NEC	dNEC	no NEC	dNEC
**0–8**	62% (7)	68% (10)	58% (5)	64% (9)	40% (8)	44% (8)	0.30 (7)	0.29 (8)	0.38 (5)	0.31 (8)	0.57 (8)	0.53(6)
**8–16**	68% (12)	67% (14)	48% (9)	63% (11)	42% (11)	53% (13)	0.26 (12)	0.28 (14)	0.46 (9)	0.35 (11)	0.53 (11)	0.45 (13)
**16–24**	64%(13)	70% (15)	56% (8)	63% (12)	39% (13)	60% (11)	0.32 (13)	0.27 (14)	0.41 (8)	0.35 (12)	0.61 (13)	0.37 (10)
**24–32**	65%(13)	72% (16)	59% (8)	55% (13)	40% (13)	56%* (10)	0.29 (13)	0.25 (16)	0.40 (8)	0.44 (13)	0.56 (13)	0.43 (10)
**32–40**	65% (13)	72% (16)	49% (9)	59% (13)	38% (12)	55%* (13)	0.30 (13)	0.26 (16)	0.49 (9)	0.39 (13)	0.61 (12)	0.40* (13)
**40–48**	64% (12)	73% (15)	53% (7)	57% (12)	40% (10)	52%* (13)	0.30 (12)	0.25 (15)	0.45 (7)	0.41 (12)	0.57 (10)	0.42* (13)

In Table 3 we present the courses of rSO_2_ and FTOE values during the first eight hours after onset of NEC symptoms. Again we found no significant differences between the two groups during these first hours after onset of NEC symptoms.

**Table 3:** RSO_2_ and FTOE values in the first eight hours after onset of NEC symptoms in preterm infants with no NEC and definite NEC. Only infants with measurements at all 3 locations are included in the analyses. Data are expressed as median values with the number of infants studied between brackets. There were no statistical differences between the two groups.

Hours	r_c_SO_2_		r_liv_SO_2_		r_int_SO_2_		cFTOE		livFTOE		intFTOE	
	no NEC	dNEC	no NEC	dNEC	no NEC	dNEC	no NEC	dNEC	no NEC	dNEC	no NEC	dNEC
**0–1**	-	-	-	-	-	-	-	-	-	-	-	-
**1–2**	-	47% (1)	-	52% (1)	-	49% (1)	-	-	-	-	-	-
**2–3**	64% (2)	47% (1)	67% (2)	55% (1)	52% (1)	44% (1)	0.31 (2)	0.49 (1)	0.28 (2)	0.41 (1)	0.37 (2)	0.55 (1)
**3–4**	62% (2)	59% (1)	67% (2)	51% (1)	59% (2)	40% (1)	0.31 (2)	0.38 (1)	0.26 (2)	0.46 (1)	0.35 (2)	0.57 (1)
**4–5**	64% (3)	59% (3)	64% (3)	76% (1)	64% (2)	70% (2)	0.30 (3)	0.36 (3)	0.26 (3)	0.22 (1)	0.27 (2)	0.26 (2)
**5–6**	59% (3)	62% (6)	52% (3)	73% (5)	42% (3)	66% (4)	0.31 (3)	0.34 (5)	0.40 (3)	0.23 (5)	0.52 (3)	0.29 (4)
**6–7**	60% (5)	67% (7)	63% (4)	63% (7)	45% (5)	48% (6)	0.32 (5)	0.13 (7)	0.30 (4)	0.32 (7)	0.51 (5)	0.42 (6)
**7–8**	60% (5)	71% (6)	56% (6)	56% (7)	50% (4)	42% (6)	0.31 (5)	0.30 (5)	0.39 (6)	0.35 (6)	0.46 (4)	0.58 (5)

### NIRS measurements in infants with uncomplicated and complicated NEC

Ten out of twenty infants with definite NEC developed complicated NEC and the other ten infants developed NEC without complications. Of the infants with complicated NEC two were diagnosed with Bell’s stage 3A. Both died as a consequence of NEC five and 35 days after onset of the symptoms. The other eight infants were found to have a perforation (Bell’s stage 3B). Seven infants were operated on with a median time of thirty-three hours (range 9–165) between onset of NEC symptoms and surgery. The other infant was too unstable clinically for surgery. Five infants were taken for surgery during the study period. NIRS monitoring in these infants was stopped after a median of ten hours (range 5–33) after onset of NEC symptoms. Of the infants with a perforation, five died as a consequence of NEC. Ischemic necrosis was confirmed by our pathologist using tissue macroscopy and microscopy in all infants with Bell’s stage 3B.

In Table 4 we provide the patient characteristics of infants with an uncomplicated and a complicated course of NEC. Infants with complicated NEC were significantly younger, received inotropes more often, underwent surgery more often, and had a higher mortality rate than infants with uncomplicated NEC.

**Table 4:** Patient characteristics of infants with uncomplicated and complicated NEC. Data are expressed as median (range) or as numbers unless otherwise specified. Abbreviations: NEC—necrotizing enterocolitis; NIRS—near-infrared spectroscopy; PDA—patent ductus arteriosus; PNA—postnatal age; RBC—red blood cell. Circulatory failure was defined as hemodynamic instability and scored by the need for volume expansion or the use of inotropes or both, from 1 hour before NEC onset until the first forty-eight hours after NEC onset or until surgery took place, whichever came first. Differences between the two groups are marked by * (< .05).

	Uncomplicated NEC (n = 10)	Complicated NEC (n = 10)
Gestational age (weeks)	30.9 (25.7–35.9)	27.2 (25.0–34.0)*
Birth weight (grams)	1518 (740–2400)	1035 (790–2280)
Male:Female	6:4	8:2
PNA at first NIRS measurement (days)	10 (3–41)	10 (7–22)
Anemia (%)	3 (30)	3 (30)
Thrombocytopenia (%)	1 (10)	4 (40)
Metabolic acidosis (%)	1 (11) (n = 9)	2 (20)
Mechanical ventilation (%)	2 (20)	4 (40)
Treated PDA before onset study (%)	1 (10)	4 (40)
PDA during study (%)	1 (10)	4 (40)
Hemodynamically significant	0	2
RBC transfusion (%)	3 (30)	4 (40)
Circulatory failure		
Fluid resuscitation (%)	4 (40)	8 (80)
Inotropes (%)	0 (-)	6 (60)*
Surgery (%)	1 (10)	8 (80)*
Death (%)	0 (-)	7 (70)*

We present the courses of rSO_2_ and FTOE values in Table 5. Preterm infants with complicated NEC had significantly lower median r_c_SO_2_ values throughout the entire study period and significantly higher cFTOE values from eight hours onwards. Furthermore, we found lower r_liv_SO_2_ and higher livFTOE values in preterm infants with complicated NEC than in infants with uncomplicated NEC in three time periods (0–8 hours, 24–32 hours, and 40–48 hours). Finally, r_int_SO_2_ was significantly lower and intFTOE higher between eight and sixteen hours and r_int_SO_2_ significantly higher and intFTOE lower between twenty-four and thirty-two hours after onset of NEC symptoms. When we analyzed only those infants who were included within eight hours after onset of NEC symptoms and in whom NIRS measurements were performed at all three locations, the direction of the results was quite similar, although significance was now mainly reached during the first eight hours and between eight and sixteen hours (S2 Table).

**Table 5:** RSO_2_ and FTOE values in the first forty-eight hours after onset of NEC symptoms in preterm infants with uncomplicated and complicated NEC. Data are expressed as median values with the number of infants studied between brackets. Statistical differences between the two groups are marked by * (< .05).

Hours	r_c_SO_2_		r_liv_SO_2_		r_int_SO_2_		cFTOE		livFTOE		intFTOE	
	unNEC	cNEC	unNEC	cNEC	unNEC	cNEC	unNEC	cNEC	unNEC	cNEC	unNEC	cNEC
**0–8**	83% (5)	65%* (5)	69% (5)	37%* (4)	77% (3)	43% (5)	0.13 (5)	0.42 (3)	0.28 (5)	0.45* (3)	0.19 (3)	0.62 (3)
**8–16**	81% (7)	55%* (7)	76% (7)	44% (4)	70% (6)	32%* (7)	0.17 (7)	0.38* (7)	0.22 (7)	0.53 (4)	0.27 (6)	0.54* (7)
**16–24**	81% (8)	54%* (7)	67% (8)	42% (4)	61% (7)	51% (4)	0.17 (8)	0.37* (6)	0.31 (8)	0.59 (4)	0.36 (7)	0.42 (3)
**24–32**	78% (9)	58%* (7)	60% (8)	31%* (5)	54% (8)	66%* (2)	0.21 (9)	0.35* (7)	0. 37 (8)	0.64* (5)	0.44 (8)	0.32* (2)
**32–40**	73% (10)	59%* (6)	59% (9)	53% (4)	48% (9)	59% (4)	0.22 (10)	0.35* (6)	0.39 (9)	0.44 (4)	0.48 (9)	0.38 (4)
**40–48**	75% (10)	55%*(5)	62% (9)	39%*(3)	47% (9)	55% (4)	0.24 (10)	0.38* (5)	0.37 (9)	0.59* (3)	0.53 (9)	0.40 (4)

In Table 6 we present the courses of rSO_2_ and FTOE values during the first eight hours after onset of NEC symptoms. Significant differences were found between the two groups during these first hours after onset of NEC symptoms in some one-hour periods but not in all. Particularly we found considerably lower r_liv_SO_2_ and r_int_SO_2_ and higher livFTOE and intFTOE values in preterm infants with complicated NEC compared with infants with uncomplicated NEC.

**Table 6:** RSO_2_ and FTOE values in the first eight hours after onset of NEC symptoms in preterm infants in preterm infants with uncomplicated and complicated NEC. Data are expressed as median values with the number of infants studied between brackets. Statistical differences between the two groups are marked by * (< .05).

Hours	r_c_SO_2_		r_liv_SO_2_		r_int_SO_2_		cFTOE		livFTOE		intFTOE	
	unNEC	cNEC	unNEC	cNEC	unNEC	cNEC	unNEC	cNEC	unNEC	cNEC	unNEC	cNEC
**0–1**	-	-	-	-	-	-	-	-	-	-	-	-
**1–2**	-	47% (1)	-	52% (1)	-	49% (1)	-	-	-	-	-	-
**2–3**	77% (1)	47% (1)	68% (1)	55% (1)	-	44% (1)	0.22 (1)	0.49 (1)	0.32 (1)	0.41 (1)	-	0.55 (1)
**3–4**	76% (1)	59% (1)	58% (1)	51% (1)	-	40% (1)	0.23 (1)	0.38 (1)	0.41 1)	0.46 (1)	-	0.57 (1)
**4–5**	78% (2)	55% (2)	65% (2)	-	83% (1)	57% (1)	0.20 (2)	0.40 (2)	0.33 (2)	-	0.14 (1)	0.38 (1)
**5–6**	74% (4)	64% (3)	73% (5)	50% (1)	80% (3)	33% (1)	0.23 (4)	0.39 (3)	0.23 (5)	0.49 (1)	0.17 (5)	0.67 (1)
**6–7**	82% (5)	61% (3)	74% (5)	27%* (3)	70% (3)	19% (3)	0.13 (5)	0.32 (3)	0.25 (5)	0.46* (3)	0.24 (3)	0.64 (3)
**7–8**	78% (4)	67% (4)	67% (5)	17% (3)	76% (3)	42% (4)	0.20 (4)	0.30 (3)	0.29 (5)	0.62 (2)	0.20 (3)	0.58 (3)

**Fig 2:**
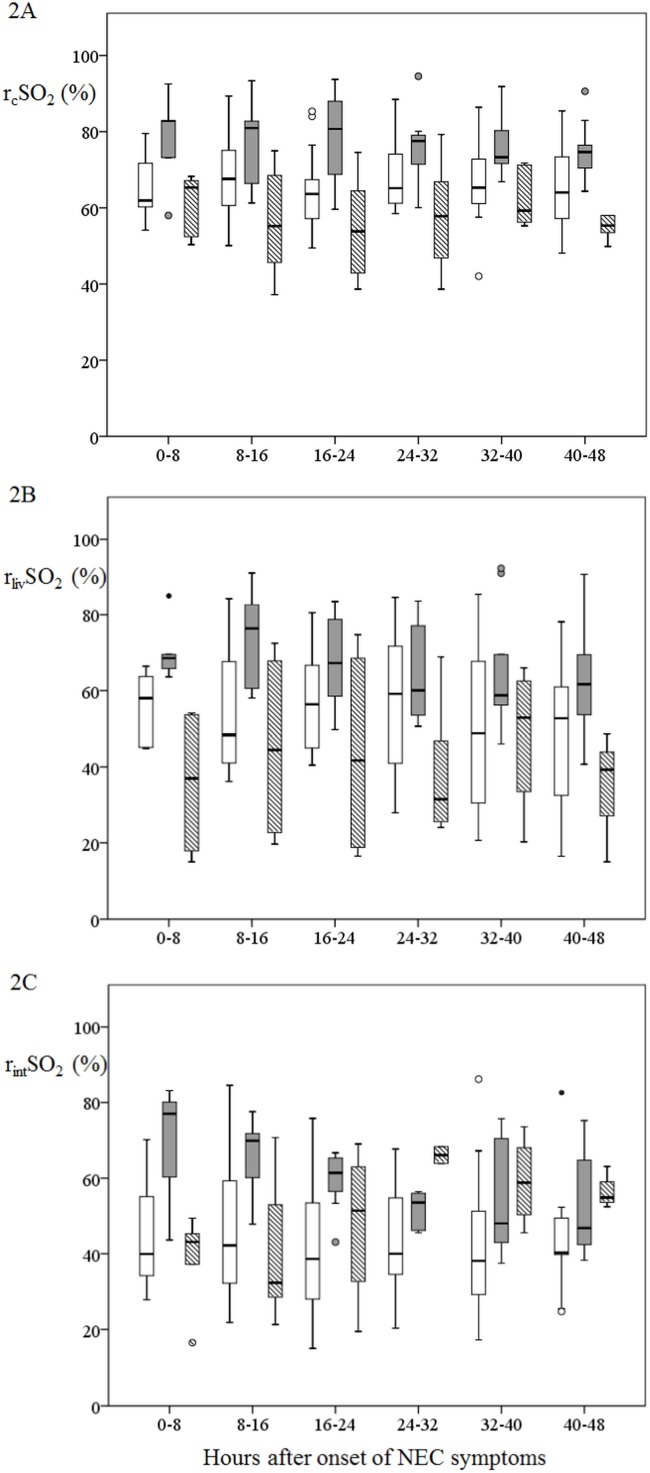
R_c_SO_2_, r_liv_SO_2_, and r_int_SO_2_ values in infants with no NEC, uncomplicated NEC, and complicated NEC. Data are shown in box and whisker plots. Dots and stars represent outliers. NoNEC is designated with a plain white boxes; uncomplicated NEC is designated with plain grey boxes, complicated NEC is designated with boxes with grey and white stripes.

In Fig 2 we present the courses of the cerebral and splanchnic rSO_2_ values in the first forty-eight hours after onset of NEC symptoms in infants with no NEC, uncomplicated NEC, and complicated NEC separately. The graphic representation of only those infants who were included within eight hours after onset of NEC symptoms and in whom NIRS measurements were performed at all three locations is shown in S1 Fig.

### ROC curves

We generated ROC curves for r_c_SO_2_ and r_liv_SO_2_ to differentiate between infants with uncomplicated and complicated NEC, since only these values showed significant differences between the groups in the first eight hours after onset of NEC symptoms. The area under the r_c_SO_2_ ROC curve was 0.88 (95% confidence interval (CI) 0.64–1.00, *P* = .047) and the area under the r_liv_SO_2_ curve was 1.00 (CI 1.00–1.00, *P* = .014). Taking a threshold value for r_c_SO_2_ of 71%, r_c_SO_2_ detected the presence of complications with a sensitivity of 1.00 (CI 0.46–1.00) and specificity of 0.80 (CI 0.30–0.99). Taking a threshold value for r_liv_SO_2_ of 59%, r_liv_SO_2_ detected the presence of complications with a sensitivity of 1.00 (CI 0.40–1.00) and specificity of 1.00 (CI 0.46–1.00).

### Variability

In Table 7 we present the intraindividual variability. Variability measurements were neither significantly different within twenty-four hours after onset of NEC symptoms between infants with no NEC and definite NEC, nor between infants with uncomplicated and complicated NEC. Between twenty-four and forty-eight hours, however, infants with definite NEC had significantly lower variability of r_int_SO_2_ measurements than infants with no NEC. Moreover, the infants with complicated NEC had a significantly higher variability of r_c_SO_2_ and lower variability of r_int_SO_2_ measurements than infants with uncomplicated NEC.

**Table 7:** Intraindividual variability of preterm infants with no NEC versus preterm infants with definite NEC, and of preterm infants with uncomplicated NEC versus preterm infants with complicated NEC. Data are expressed as median (range). Abbreviations: NEC, necrotizing enterocolitis; r_c_SO_2_, cerebral tissue oxygen saturation; r_liv_SO_2_, liver tissue oxygen saturation; r_int_SO_2_, infraumbilical tissue oxygen saturation. Intraindividual variability is defined as the daily percentage of time that one-hour mean rSO_2_ values were 15% or more below or above the infant’s daily mean.

		No NEC	Definite NEC	*P* value	Uncomplicated NEC	Complicated NEC	*P* value
**0–24 hr**	**r**_**c**_**SO**_**2**_ **(%)**	0 (0–39)	0 (0–14)	.106	0 (0–6)	0 (0–14)	.433
	**r**_**liv**_**SO**_**2**_ **(%)**	21 (0–44)	6 (0–42)	.421	12 (0–42)	6 (0–6)	.302
	**r**_**int**_**SO**_**2**_ **(%)**	16 (0–48)	15 (0–55)	.667	15 (0–55)	12 (0–36)	.999
**24–48 hr**	**r**_**c**_**SO**_**2**_ **(%)**	0 (0–14)	0 (0–33)	.951	0 (0–0)	4 (0–33)	.017*
	**r**_**liv**_**SO**_**2**_ **(%)**	13 (4–67)	13 (0–86)	.421	13 (4–86)	9 (0–25)	.393
	**r**_**int**_**SO**_**2**_ **(%)**	22 (0–78)	4 (0–25)	.022*	17 (0–25)	0 (0–4)	.022*

* Indicates *P* < .05.

## Discussion

Our study suggests that NIRS monitoring can be useful in preterm infants with definite NEC to differentiate in the first eight hours after onset of symptoms between those infants who would develop complicated NEC and those who would not. The low oxygen saturation values and high oxygen extraction values of splanchnic and cerebral tissue are associated with the progression to a bowel perforation or death. Conversely, the data indicate that, in our sample, NIRS monitoring did not help to differentiate between infants with definite NEC and infants who were diagnosed differently, in the early stages of the disease with clinical signs pointing to NEC.

Our first aim was to determine whether NIRS monitoring was able to discriminate between definite NEC (Bell’s stages 2 & 3) and no NEC (Bell’s stage 1 at most), at the onset of symptoms suggestive of NEC. In contrast to our hypothesis, we did not find significant differences between infants with definite NEC and infants with no NEC during the first twenty-four hours after onset of NEC symptoms. Stapleton et al. described a similar course of splanchnic NIRS measurements in an infant with an uncomplicated course of NEC, showing improvement of NIRS values after approximately twenty-four hours [5]. Our findings seem to be in contrast, however, to those of Fortune et al. [3]. They reported that the simultaneous measurement of brain and splanchnic NIRS was able to discriminate whether or not splanchnic ischemia was present in infants with an acute abdomen. They included, however, only infants (n = 5) with complicated NEC, all of them requiring surgery. Their splanchnic NIRS values are in the same range as in our infants with complicated NEC. Moreover, their control group is quite different in nature than ours, consisting of infants with very heterogeneous diagnoses. In their report, the infants with diagnoses that go along with circulatory failure (sepsis, persistent ductus arteriosus, coarctation) also have rather low splanchnic NIRS values, similar as our controls. We believe therefore that in our study the underlying conditions which were finally diagnosed in preterm infants with no NEC, such as volvulus and sepsis, may have had similar effects on the splanchnic rSO_2_ and FTOE values obtained with NIRS as those observed in NEC. The higher prevalence of anemia in infants without NEC might also have contributed to the lower rSO_2_ values in this group, since a low concentration of hemoglobin corresponds to lower oxygen saturation values [11].

Our second aim was to determine the value of splanchnic NIRS monitoring to predict a complicated course in preterm infants with definite NEC. In the first twenty-four hours after NEC onset, we demonstrated that both splanchnic and cerebral oxygen saturations were lower and that splanchnic and cerebral oxygen extractions were higher in preterm infants who developed complicated NEC. This is in line with the findings of Fortune at al [3], which we now confirm in a larger group of infants with a broad variety of severity of NEC. They are also consistent with a case report by Zabaneh et al, who reported on preterm twins, one with a complicated NEC, and the other without NEC [4]. They described that mesenteric NIRS was low in the twin with NEC, forty-eight hours after onset of the disease, the location corresponding with necrotic bowel found at surgery [4]. We add to their findings that the splanchnic low rSO_2_ and high FTOE values are already present during the first eight to sixteen hours after onset of NEC symptoms.

We offer several explanations for these findings. First, blood flow to the splanchnic bed may be reduced due to the presence of ischemic/necrotic bowel. This explanation is supported by a study of a pig model of NEC, reporting low mesenteric NIRS values for several days in those animals who go on to develop ischemic and necrotic bowel [12]. A second explanation relates to illness severity. This might have been so severe in those infants who developed complicated NEC that circulatory insufficiency ensued in the early stage of NEC. Perfusion to less essential organs, such as the intestine, will be affected first [13]. When insufficiency becomes more severe, however, the cerebral perfusion will also be compromised [13]. Indeed, cerebral oxygen saturation was lower and extraction was higher in preterm infants with complicated NEC than in preterm infants with uncomplicated NEC.

A third explanation would be that preterm infants with uncomplicated NEC might have had higher splanchnic oxygen saturation and lower oxygen extraction values due to a relatively increased intestinal blood flow compared to preterm infants with complicated NEC, caused by the inflammatory response seen in NEC. Increased blood flow velocities in the superior mesenteric artery and the celiac axis, the major contributors of blood flow to the intestinal tissue, have been shown in preterm infants with NEC compared to preterm infants without abdominal disease [14, 15]. Moreover, McNeill et al. reported infraumbilical saturation values of 35% to 55% ten days after birth for relatively stable preterm infants between 29 to 33 weeks of gestation [10]. We found a higher median saturation level of 77% in the first eight hours after onset of NEC symptoms in preterm infants with uncomplicated NEC.

Finally, the younger gestational age of infants with complicated NEC in comparison to infants with uncomplicated NEC might have contributed to the differences we found for splanchnic rSO_2_ and FTOE values between these two groups [10]. This assumption, however, is based on a study performed in relatively healthy preterm infants who were fed normally [10]. The effect of NEC and its treatment on splanchnic oxygen saturation values makes an interpretation of the effect of gestational age on these values difficult, if not impossible.

Regarding variability measurements, we did not find any significant differences between preterm infants with suspected and definite NEC, and between infants with complicated and uncomplicated NEC in the first twenty-four hours after onset of NEC symptoms. Although Cortez et al. suggested that loss of variability might be helpful to predict the onset of NEC our study suggests that these measurements might not be useful once NEC is suspected or diagnosed [16].

In this study we have shown that values of r_c_SO_2_ ≤ 71% and r_liv_SO_2_ ≤ 59% during the first eight hours after onset of symptoms predicted complicated NEC in infants with definite NEC, with a sensitivity of 100% and specificity of 80% and sensitivity of 100% and specificity of 100%, respectively. These results suggest that monitoring cerebral and splanchnic rSO_2_ might be helpful in clinical practice in predicting the course of NEC. However, we would like to stress the fact that these findings are based on measurements performed in a small sample size. Additionally, we did not control for potential confounders, such as gestational age and vasopressor medications. Our results, therefore, warrant further research in a larger patient population before we can be confident of its usefulness.

We acknowledge several limitations to our study. First of all, we included a relatively small sample size. Second, sensor replacement could unintentionally have caused variability in rSO_2_ measurements. We do believe, however, that this phenomenon was distributed evenly over the various subgroups analyzed. Third, we did not include every preterm infant within eight hours after onset of NEC symptoms and we were unable to measure the entire forty-eight hours in each infant which may have caused a sampling error. Finally, the splanchnic NIRS measurements might have been influenced by air, stools, movements of the gut within the abdominal cavity, and peristaltic movements [3, 17].

Our study might have implications. Our findings suggested that NIRS did not serve the purpose of being able to distinguish between NEC and other intestinal diseases during the early stages of the disease. However, NIRS was able to alert the clinician that bowel ischemia was present, indicating a complicated course of NEC already within the first eight to sixteen hours after onset of NEC symptoms. This may be an important clinical finding because early recognition of patients in need for surgery could benefit the patient.

## Conclusions

Our findings suggest that monitoring oxygen saturation and extraction at the cerebral and splanchnic region in preterm infants can help us to differentiate between complicated and uncomplicated NEC. However, in our sample we found no relevant added-value for NIRS in the early diagnostic process of preterm infants with clinical signs suspicious of NEC.

## Supporting Information

S1 Table Alternative Table 2. RSO_2_ and FTOE values in the first forty-eight hours after onset of NEC symptoms in preterm infants with no NEC and definite NEC, in the left of each cell all measurements, in the right only measurements of children included <8h after onset and available NIRS values at all three locations. Data are expressed as median values with the number of infants studied between brackets. Statistical differences between the two groups are marked by * (< .05).
(DOC)

S2 Table Alternative Table 5. RSO_2_ and FTOE values in the first forty-eight hours after onset of NEC symptoms in preterm infants with uncomplicated and complicated NEC, in the left of each cell all measurements, in the right only measurements of children included <8h after onset and available NIRS values at all **three locations.** Data are expressed as median values with the number of infants studied between brackets. Statistical differences between the two groups are marked by * (< .05).
(DOC)

S1 Fig Alternative Fig 2. R_c_SO_2_, r_liv_SO_2_, and r_int_SO_2_ values in infants with no NEC, uncomplicated NEC, and complicated NEC, presenting only measurements of children included <8h after onset and available NIRS values at all three locations. Data are shown in box and whisker plots. Dots and stars represent outliers. NoNEC is designated with blue boxes; uncomplicated NEC is designated with green boxes, complicated NEC is designated with grey boxes.
(JPG)
