# Supplementary material for: Near-Infrared Spectroscopy to Predict the Course of Necrotizing Enterocolitis
Source: PLoS One. 2016 May 16;11(5):e0154710. doi: 10.1371/journal.pone.0154710 (PMC4868291; doi:10.1371/journal.pone.0154710)
Supplement: S2 Table — RSO2 and FTOE values in the first forty-eight hours after onset of NEC symptoms in preterm infants with uncomplicated and complicated NEC, in the left of each cell all measurements, in the right only measurements of children included <8h after onset and available NIRS values at all three locations. Data are expressed as median values with the number of infants studied between brackets. Statistical differences between the two groups are marked by * (< .05). (DOC) [file pone.0154710.s002.doc]

**S2 Table. Alternative Table 4A. RSO_2_ and FTOE values in the first forty-eight hours after onset of NEC symptoms in preterm infants with uncomplicated and complicated NEC, in the left of each cell all measurements, in the right only measurements of children included <8h after onset and available NIRS values at all three locations**.

| **Hours** | **r_c_SO_2_** | | **r_liv_SO_2_** | | **r_int_SO_2_** | | **cFTOE** | | **livFTOE** | | **intFTOE** | |
| --- | --- | --- | --- | --- | --- | --- | --- | --- | --- | --- | --- | --- |
|  | **unNEC** | **cNEC** | **unNEC** | **cNEC** | **unNEC** | **cNEC** | **unNEC** | **cNEC** | **unNEC** | **cNEC** | **unNEC** | **cNEC** |
| **0-8** | 83% 83%  (5) (4) | 65%* 60%  (5) (4) | 69% 69%  (5) (4) | 37%* 37%*  (4) (4) | 77% 77%  (3) (3) | 43% 40%  (5) (4) | 0.13 0.13  (5) (4) | 0.42 0.43  (3) (3) | 0.28 0.27  (5) (4) | 0.45* 0.45  (3) (3) | 0.19 0.19  (3) (3) | 0.62 0.62  (3) (3) |
| **8-16** | 81% 82%  (7) (4) | 55%* 46%  (7) (3) | 76% 69%  (7) (4) | 44% 26%  (4) (3) | 70% 65%  (6) (4) | 32%* 31%  (7) (3) | 0.17 0.15  (7) (4) | 0.38* 0.45  (7) (3) | 0.22 0.29  (7) (4) | 0.53 0.71  (4) (3) | 0.27 0.33  (6) (4) | 0.54* 0.54  (7) (3) |
| **16-24** | 81% 85%  (8) (4) | 54%* 60%  (7) (2) | 67% 72%  (8) (4) | 42% 48%  (4) (2) | 61% 61%  (7) (4) | 51% 38%  (4) (2) | 0.17 0.14  (8) (4) | 0.37* 0.40  (6) (2) | 0.31 0.27  (8) (4) | 0.59 0.53  (4) (2) | 0.36 0.37  (7) (4) | 0.42 0.54  (3) (2) |
| **24-32** | 78% 80%  (9) (4) | 58%* 59%  (7) (2) | 60% 71%  (8) (4) | 31%* 50%  (5) (2) | 54% 54%  (8) (4) | 66%* 68%  (2) (1) | 0.21 0.20  (9) (4) | 0.35* 0.37  (7) (2) | 0.37 0.29  (8) (4) | 0.64* 0.47  (5) (2) | 0.44 0.44  (8) (4) | 0.32* 0.30  (2) (1) |
| **32-40** | 73% 80% (10) (4) | 59%* 63%  (6) (2) | 59% 74%  (9) (4) | 53% 63%  (4) (2) | 48% 67%  (9) (4) | 59% 64%  (4) (2) | 0.22 0.19 (10) (4) | 0.35* 0.34  (6) (2) | 0.39 0.25  (9) (4) | 0.44 0.34  (4) (2) | 0.48 0.31  (9) (4) | 0.38 0.33  (4) (2) |
| **40-48** | 75% 80% (10) (4) | 55%* 54%  (5) (2) | 62% 69%  (9) (4) | 39%* 49%  (3) (2) | 47% 54%  (9) (4) | 55% 55%  (4) (2) | 0.24 0.18 (10) (4) | 0.38* 0.40  (5) (2) | 0.37 0.29  (9) (4) | 0.59* 0.45  (3) (1) | 0.53 0.44  (9) (4) | 0.40 0.40  (4) (2) |

Data are expressed as median values with the number of infants studied between brackets.

Statistical differences between the two groups are marked by * (< .05).
