# Supplementary figures and images for: Near-Infrared Spectroscopy to Predict the Course of Necrotizing Enterocolitis
Source: PLoS One. 2016 May 16;11(5):e0154710. doi: 10.1371/journal.pone.0154710 (PMC4868291; doi:10.1371/journal.pone.0154710)

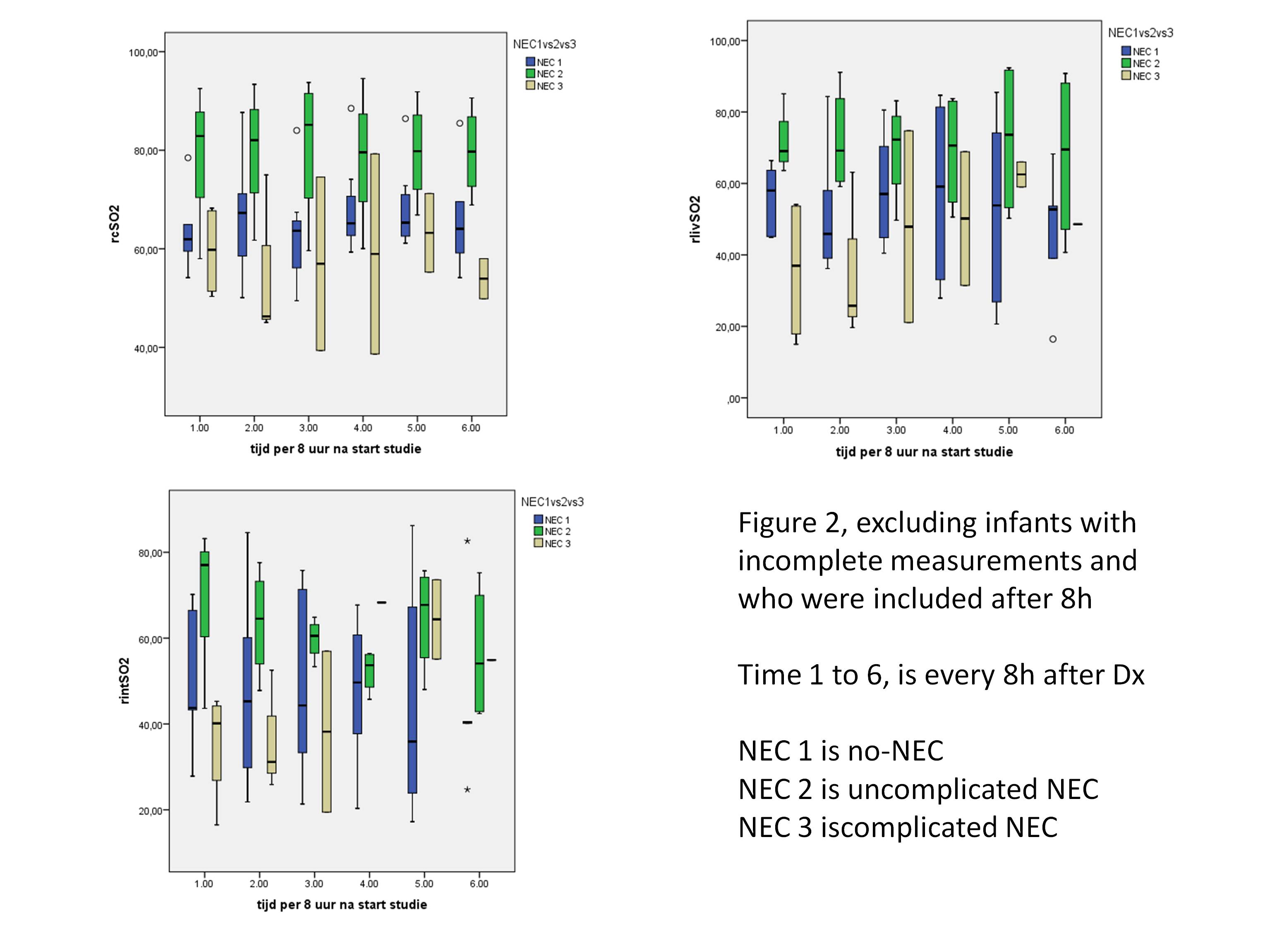

Supplement: S1 Fig — RcSO2, rlivSO2, and rintSO2 values in infants with no NEC, uncomplicated NEC, and complicated NEC, presenting only measurements of children included <8h after onset and available NIRS values at all three locations. Data are shown in box and whisker plots. Dots and stars represent outliers. NoNEC is designated with blue boxes; uncomplicated NEC is designated with green boxes, complicated NEC is designated with grey boxes. (JPG) [file pone.0154710.s003.jpg]
